# Chloridotetra­kis(pyridine-4-carb­alde­hyde-κ*N*)copper(II) chloride

**DOI:** 10.1107/S1600536809041816

**Published:** 2009-10-17

**Authors:** Xiu-Jin Meng, Shu-Hua Zhang, Ge-Ge Yang, Xue-Ren Huang, Yi-Min Jiang

**Affiliations:** a College of Chemistry and Chemical Engineering, Guangxi Normal University, Guilin, Guangxi 541004, People’s Republic of China; b Key Laboratory of New Processing Technology for Nonferrous Metals & Materials, Ministry of Education, Guilin University of Technology, Guilin, Guangxi 541004, People’s Republic of China

## Abstract

In the mol­ecular structure of the title compound, [CuCl(C_6_H_5_NO)_4_]Cl, the Cu^II^ atom is coordinated by four N atoms of four pyridine-4-carboxaldehyde ligands and one chloride anion in a slightly distorted square-pyramidal coordination geometry. There is also a non-coordinating Cl^−^ anion in the crystal structure. The Cu^II^ atom and both Cl atoms are situated on fourfold rotation axes. A weak C—H⋯Cl inter­action is also present.

## Related literature

For other compounds with pyridine-4-carbaldehyde ligands, see: Rivera & Sheldrick (1977 ▶); Choi & Wong (1999 ▶); Briand *et al.* (2007 ▶); Sie *et al.* (2008 ▶). 
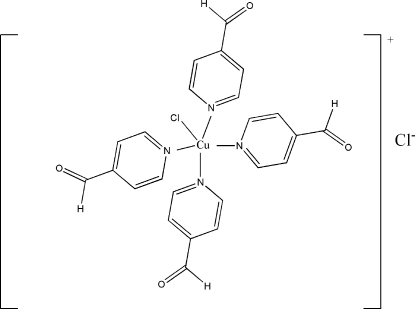


## Experimental

### 

#### Crystal data

[CuCl(C_6_H_5_NO)_4_]Cl
*M* *_r_* = 562.88
Tetragonal, 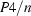

*a* = 10.5035 (3) Å
*c* = 11.3751 (6) Å
*V* = 1254.94 (8) Å^3^
*Z* = 2
Mo *K*α radiation
μ = 1.12 mm^−1^
*T* = 296 K
0.38 × 0.21 × 0.18 mm

#### Data collection

Bruker SMART CCD area-detector diffractometer
Absorption correction: multi-scan (*SADABS*; Sheldrick, 1996 ▶) *T*_min_ = 0.675, *T*_max_ = 0.825
9150 measured reflections
1126 independent reflections
1083 reflections with *I* > 2σ(*I*)
*R*_int_ = 0.017

#### Refinement

*R*[*F* ^2^ > 2σ(*F* ^2^)] = 0.033
*wR*(*F* ^2^) = 0.114
*S* = 1.03
1126 reflections
82 parameters
13 restraints
H-atom parameters constrained
Δρ_max_ = 0.56 e Å^−3^
Δρ_min_ = −0.48 e Å^−3^

### 

Data collection: *SMART* (Bruker, 2004 ▶); cell refinement: *SAINT* (Bruker, 2004 ▶); data reduction: *SAINT*; program(s) used to solve structure: *SHELXS97* (Sheldrick, 2008 ▶); program(s) used to refine structure: *SHELXL97* (Sheldrick, 2008 ▶); molecular graphics: *SHELXTL* (Sheldrick, 2008 ▶); software used to prepare material for publication: *SHELXTL*.

## Supplementary Material

Crystal structure: contains datablocks I, global. DOI: 10.1107/S1600536809041816/im2137sup1.cif

Structure factors: contains datablocks I. DOI: 10.1107/S1600536809041816/im2137Isup2.hkl

Additional supplementary materials:  crystallographic information; 3D view; checkCIF report

## Figures and Tables

**Table 1:** Hydrogen-bond geometry (Å, °)

*D*—H⋯*A*	*D*—H	H⋯*A*	*D*⋯*A*	*D*—H⋯*A*
C5—H5⋯Cl2	0.93	2.84	3.732 (2)	160

**Fig. 1.**
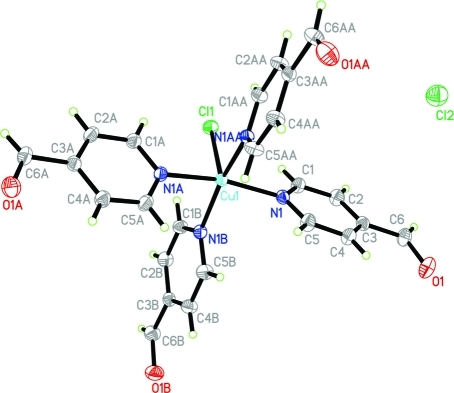
Ellipsoid plot (30% probability) of the title compound showing the numbering scheme. Dashed lines indicate hydrogen bonds. Symmetry code: 1# -x + 1/2, y + 1/2, z.

**Fig. 2.**
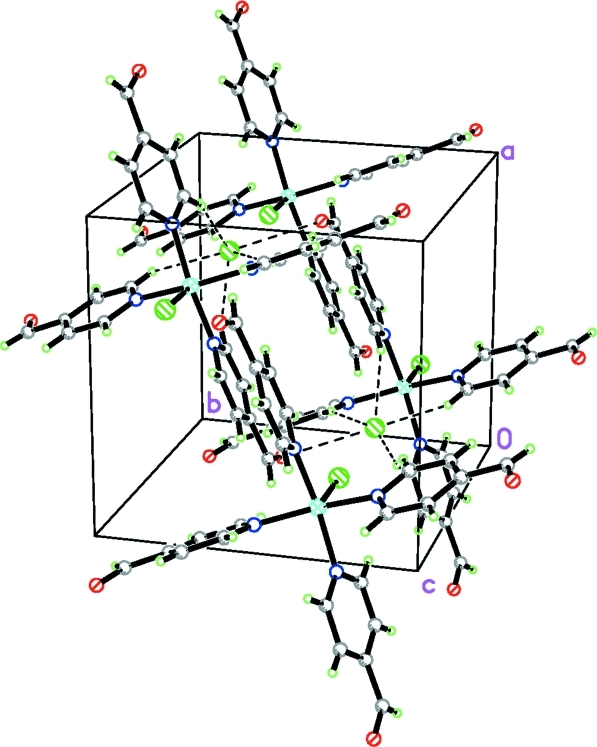
2-D network, as viewed down the c axis. Dashed lines indicate hydrogen bonds.

## supplementary crystallographic information

### Comment

Only one structurally characterized coordination compound with pyridine-4- carboxaldehyde acting as the ligand has been reported up to now. In that article, pyridine-4-carboxaldehyde and CoBr_2_ form [CoBr_2_(C_5_H_4_N-CHO)_4_] (Rivera *et al.* 1977). This compound is highly related to the title compound. In addition, three crystal structures with pyridine-4-carboxaldehyde acting as independent components were reported (Choi *et al.* 1999; Briand *et al.* 2007; Sie *et al.* 2008).

In the cation of the title compound [CuCl(OCHC_5_H_4_N)_4_]Cl, the Cu^II^ centre is coordinated to four N atoms from four pyridine-4-carboxaldehyde ligands and one chloro ligand. Cu exhibits a slightly distorted square-pyramidal coordination geometry. Another non-coordinating chloride anion is observed in the crystal structure. The [CuCl(C_5_H_4_N-CHO)_4_]^+^ ion has a perfect *C_4_* symmetry with the direction of the *C_4_* axis being collinear with the Cu1—Cl1 direction. Cu1, Cl1 and Cl2 are all situated on the same crystallographic 4-fold rotoinversion axis. In the cation therefore all Cu—N bond lengths and angles are equivalent.

Several donor CH functions and the chloride acceptor groups participate in the observed hydrogen bonding pattern forming a two-dimensional network in the *ab* plane (Fig. 2)

### Experimental

For the preparation of the title compound, a solution of CuCl_2_ × 2 H_2_O (0.08524 g, 0.5 mmol) in H_2_O(5 ml) was slowly added over a period of 2 h to a solution of *L*-Cysteic acid (0.094 g, 0.5 mmol), KOH (0.056 g, 1 mmol), pyridine-4-carboxaldehyde (0.06 ml, 0.6 mmol) and NaBH_4_ (0.03028 g, 0.8 mmol) in methanol (20 ml) resulting in a blue solution that was stirred for another 4 h at 298 K. Then, the solution was left to evaporate slowly at room temperature. After ten days, blue block crystals of the title compoound were obtained with a yield of 70%.

### Refinement

H atom bonded to C atom were positioned geometrically with the C—H distance of 0.9303 Å, and treated as riding atoms, with *U*_iso_(H) = 1.2*U*_eq_(C).

### Crystal data

**Table:**

[CuCl(C_6_H_5_NO)4]Cl	*D*_x_ = 1.490 Mg m^−^^3^
*M**_r_* = 562.88	Mo *K*α radiation, λ = 0.71073 Å
Tetragonal, *P*4/*n*	Cell parameters from 1162 reflections
Hall symbol: -P 4a	θ = 2.6–25.1°
*a* = 10.5035 (3) Å	µ = 1.12 mm^−^^1^
*c* = 11.3751 (6) Å	*T* = 296 K
*V* = 1254.94 (8) Å^3^	Block, blue
*Z* = 2	0.38 × 0.21 × 0.18 mm
*F*(000) = 574	

### Data collection

**Table:**

Bruker SMART CCD area-detector diffractometer	1126 independent reflections
Radiation source: fine-focus sealed tube	1083 reflections with *I* > 2σ(*I*)
graphite	*R*_int_ = 0.017
phi and ω scans	θ_max_ = 25.1°, θ_min_ = 2.6°
Absorption correction: multi-scan (*SADABS*; Sheldrick, 1996)	*h* = −12→12
*T*_min_ = 0.675, *T*_max_ = 0.825	*k* = −12→12
9150 measured reflections	*l* = −12→13

### Refinement

**Table:**

Refinement on *F*^2^	Primary atom site location: structure-invariant direct methods
Least-squares matrix: full	Secondary atom site location: difference Fourier map
*R*[*F*^2^ > 2σ(*F*^2^)] = 0.033	Hydrogen site location: inferred from neighbouring sites
*wR*(*F*^2^) = 0.114	H-atom parameters constrained
*S* = 1.03	*w* = 1/[σ^2^(*F*_o_^2^) + (0.091*P*)^2^ + 0.4771*P*] where *P* = (*F*_o_^2^ + 2*F*_c_^2^)/3
1126 reflections	(Δ/σ)_max_ < 0.001
82 parameters	Δρ_max_ = 0.56 e Å^−^^3^
13 restraints	Δρ_min_ = −0.48 e Å^−^^3^

### Special details

**Table:**

Geometry. All e.s.d.'s (except the e.s.d. in the dihedral angle between two l.s. planes) are estimated using the full covariance matrix. The cell e.s.d.'s are taken into account individually in the estimation of e.s.d.'s in distances, angles and torsion angles; correlations between e.s.d.'s in cell parameters are only used when they are defined by crystal symmetry. An approximate (isotropic) treatment of cell e.s.d.'s is used for estimating e.s.d.'s involving l.s. planes.
Refinement. Refinement of *F*^2^ against ALL reflections. The weighted *R*-factor *wR* and goodness of fit *S* are based on *F*^2^, conventional *R*-factors *R* are based on *F*, with *F* set to zero for negative *F*^2^. The threshold expression of *F*^2^ > σ(*F*^2^) is used only for calculating *R*-factors(gt) *etc*. and is not relevant to the choice of reflections for refinement. *R*-factors based on *F*^2^ are statistically about twice as large as those based on *F*, and *R*- factors based on ALL data will be even larger.

### Fractional atomic coordinates and isotropic or equivalent isotropic displacement parameters (Å^2^)

**Table:**

	*x*	*y*	*z*	*U*_iso_*/*U*_eq_	
Cu1	0.7500	0.7500	0.81702 (4)	0.0289 (3)	
Cl1	0.7500	0.7500	1.03833 (9)	0.0373 (3)	
C1	0.8616 (2)	0.5010 (2)	0.8824 (2)	0.0376 (5)	
H1	0.8631	0.5372	0.9570	0.045*	
C2	0.9093 (3)	0.3791 (2)	0.8675 (2)	0.0418 (6)	
H2	0.9404	0.3342	0.9319	0.050*	
C3	0.9109 (2)	0.3241 (2)	0.7569 (2)	0.0342 (5)	
C4	0.8612 (3)	0.3938 (2)	0.6653 (2)	0.0411 (6)	
H4	0.8601	0.3603	0.5896	0.049*	
C5	0.8132 (3)	0.5140 (3)	0.6867 (2)	0.0422 (6)	
H5	0.7790	0.5593	0.6240	0.051*	
C6	0.9662 (3)	0.1930 (2)	0.7405 (3)	0.0466 (6)	
H6	0.9939	0.1428	0.8027	0.056*	
N1	0.81319 (18)	0.56845 (17)	0.79272 (17)	0.0320 (4)	
O1	0.9721 (2)	0.1535 (2)	0.6224 (2)	0.0598 (6)	
Cl2	0.7500	0.7500	0.44844 (12)	0.0562 (4)	

### Atomic displacement parameters (Å^2^)

**Table:**

	*U*^11^	*U*^22^	*U*^33^	*U*^12^	*U*^13^	*U*^23^
Cu1	0.0261 (3)	0.0261 (3)	0.0344 (4)	0.000	0.000	0.000
Cl1	0.0410 (4)	0.0410 (4)	0.0299 (5)	0.000	0.000	0.000
C1	0.0418 (13)	0.0357 (12)	0.0354 (11)	0.0033 (10)	−0.0029 (10)	−0.0013 (9)
C2	0.0487 (15)	0.0378 (13)	0.0390 (13)	0.0075 (11)	−0.0068 (10)	0.0066 (10)
C3	0.0288 (11)	0.0306 (11)	0.0434 (12)	−0.0011 (8)	0.0001 (9)	0.0016 (9)
C4	0.0485 (15)	0.0372 (13)	0.0377 (11)	0.0062 (11)	−0.0016 (10)	−0.0031 (10)
C5	0.0527 (16)	0.0355 (13)	0.0385 (13)	0.0079 (11)	−0.0074 (10)	0.0039 (9)
C6	0.0514 (15)	0.0348 (13)	0.0536 (15)	0.0105 (11)	−0.0040 (12)	−0.0009 (11)
N1	0.0314 (10)	0.0282 (9)	0.0364 (9)	0.0000 (7)	−0.0003 (8)	0.0025 (8)
O1	0.0654 (14)	0.0500 (12)	0.0642 (13)	0.0141 (10)	−0.0063 (10)	−0.0171 (10)
Cl2	0.0619 (6)	0.0619 (6)	0.0448 (7)	0.000	0.000	0.000

### Geometric parameters (Å, °)

**Table:**

Cu1—N1^i^	2.0380 (19)	C2—H2	0.9300
Cu1—N1	2.0380 (19)	C3—C4	1.377 (3)
Cu1—N1^ii^	2.0380 (19)	C3—C6	1.506 (3)
Cu1—N1^iii^	2.0380 (19)	C4—C5	1.381 (4)
Cu1—Cl1	2.5175 (11)	C4—H4	0.9300
C1—N1	1.342 (3)	C5—N1	1.335 (3)
C1—C2	1.385 (4)	C5—H5	0.9300
C1—H1	0.9300	C6—O1	1.407 (4)
C2—C3	1.384 (4)	C6—H6	0.9300
			
N1^i^—Cu1—N1	88.946 (16)	C4—C3—C2	117.5 (2)
N1^i^—Cu1—N1^ii^	164.41 (11)	C4—C3—C6	122.6 (2)
N1—Cu1—N1^ii^	88.946 (16)	C2—C3—C6	119.9 (2)
N1^i^—Cu1—N1^iii^	88.946 (15)	C3—C4—C5	119.4 (2)
N1—Cu1—N1^iii^	164.41 (11)	C3—C4—H4	120.3
N1^ii^—Cu1—N1^iii^	88.946 (16)	C5—C4—H4	120.3
N1^i^—Cu1—Cl1	97.79 (6)	N1—C5—C4	123.4 (2)
N1—Cu1—Cl1	97.79 (6)	N1—C5—H5	118.3
N1^ii^—Cu1—Cl1	97.79 (6)	C4—C5—H5	118.3
N1^iii^—Cu1—Cl1	97.79 (6)	O1—C6—C3	113.9 (2)
N1—C1—C2	122.2 (2)	O1—C6—H6	123.0
N1—C1—H1	118.9	C3—C6—H6	123.0
C2—C1—H1	118.9	C5—N1—C1	117.4 (2)
C3—C2—C1	120.1 (2)	C5—N1—Cu1	121.56 (16)
C3—C2—H2	120.0	C1—N1—Cu1	120.97 (16)
C1—C2—H2	120.0		

Symmetry codes: (i) *y*, −*x*+3/2, *z*; (ii) −*y*+3/2, *x*, *z*; (iii) −*x*+3/2, −*y*+3/2, *z*.

### Hydrogen-bond geometry (Å, °)

**Table:**

*D*—H···*A*	*D*—H	H···*A*	*D*···*A*	*D*—H···*A*
C5—H5···Cl2	0.93	2.84	3.732 (2)	160
